# Tackling the Ubiquity of Plastic Waste for Human and Planetary Health

**DOI:** 10.4269/ajtmh.21-0968

**Published:** 2021-11-08

**Authors:** Erika M. Veidis, A. Desiree LaBeaud, Allison A. Phillips, Michele Barry

**Affiliations:** ^1^ Stanford Center for Innovation in Global Health, Stanford University School of Medicine, Stanford, California;; ^2^ Woods Institute for the Environment, Stanford University, Stanford, California

An expanding human population, extractive and polluting industrial practices, and growing resource consumption are accelerating global environmental change, with various urgent and yet-to-be-understood health impacts. This is particularly tangible in the growing challenge of plastic pollution—now found in our oceans, our air, our neighborhoods, our food, our drinking water, and our bodies—which poses various threats to human health and the environment globally.

Since the 1950s, we have produced more than 8 billion tons of plastic, more than quadrupling global production, and have recycled less than 10% of it.^1^^,^^2^ With some plastics estimated to take more than 400 years to decompose,^1^ the accumulation of plastic waste continues to accelerate rapidly. This is especially true in low- and middle-income countries (LMICs), where, generally speaking, waste infrastructure hasn’t been able to keep up with plastic use and foreign imports. And, despite various plastic bans, as in 34 of 54 of Africa’s countries, and the threat of fines and even jail time, regulation hasn’t always been successful.^3^^–^^6^

The plastic challenge is only accelerating in many parts of the world, particularly where plastic consumption outpaces population growth. For instance, over the course of 25 years, as Nairobi’s population tripled, the city saw its plastic consumption quadruple.^7^ As petrochemical companies increasingly invest in plastic production, their search for new markets stands to exacerbate this challenge. Interest groups such as the American Chemistry Council are reportedly working to influence plastic policies in some of the African countries already grappling with plastic overload—most notably Kenya—lobbying to loosen restrictions on imports of foreign waste and sometimes reverse bans on single-use plastics.^8^

In many ways, the COVID-19 pandemic has further exacerbated the global plastics problem.^9^ Hygiene concerns have prompted a surge in demand for single-use plastics, including disposable personal protective equipment, which has made the amount of medical waste up to six times greater than usual, as well as plastic packaging from takeout meals and home-delivered groceries. Low oil prices, driven by a decrease in demand for travel across the globe, shifts in trade boundaries, and the cancellation of recycling exports, among other factors, have made it cheaper to produce new plastics from oil rather than recycle existing ones, which is particularly striking given that upward of 90% of plastics already come from virgin feedstock.^10^ Concerned with managing potentially contaminated plastics, some governments have also backtracked on single-use plastic bans.^4^

As larger plastic pieces break down, they create micro- and nanoplastics (plastic pieces less than 5 mm and 100 nm, respectively), which can also be released directly into the air and waterways through industrial processes. Some studies have found that more than 80% of tap water samples worldwide contain microplastics.^11^^,^^12^ Others estimate that we consume a credit card’s worth of plastic every week.^13^ For the first time, microplastics have also been found in the human placenta,^14^ offering new and concerning insights into how these particles can travel in the body and affect health. Whether inhaled, absorbed topically, or ingested through a wide array of water and food products—ranging from seafood to salt to vegetables to anything packaged in plastic—these tiny pieces of plastic can enter the body and affect human health in a variety of ways.^15^

Microplastic chemical additives and the heavy metals and hydrophobic pollutants they attract to their surfaces have the potential to cause immune disruption, endocrine disruption, cytotoxicity, damage to organ function, inflammation, carcinogenic effects, and oxidative stress—and thereby heighten disease risks.^15^ Bacteria have also been found to bind to plastics through biofilms, sparking questions about whether plastics can expose humans to pathogens.^15^^,^^16^ Furthermore, new findings raise concerns about health effects specific to pregnancy, including through the disruption of numerous immune, endocrine, and communications pathways, with potential impacts on preeclampsia, fetal growth restriction, and other adverse pregnancy outcomes.^14^

Although much of the discussion on plastics and health centers on micro- and nanoplastics, macro-plastics also cause harm, especially in LMICs. The lack of adequate solid waste management—and consequent storing of trash at home—in sub-Saharan Africa and other LMICs is a growing problem that, among other challenges, provides habitats for mosquito breeding and contributes to infectious disease.^17^^–^^22^ *Aedes aegypti*, which transmits arboviral diseases such as dengue, chikungunya, yellow fever, and Zika, prefers to breed in man-made containers, including plastic containers, tires, and trash.^23^^–^^26^ In addition to vector-borne diseases, plastic trash also can promote cholera, dysentery, and other diseases through providing environments for microbes^27^^,^^28^ and contaminating water sources.^29^

Plastic waste also leads to indirect health risks; when enough trash accumulates, the most common approach to eliminating it is to burn it onsite at household and community dump sites. Globally, an estimated one quarter of all plastic waste is incinerated.^4^^,^^30^ In settings with limited waste management infrastructures, this figure is even higher. In sub-Saharan Africa, for example, more than 75% of all waste, including plastics, is burned. This contributes to pollution and releases toxins into the air—including dioxins, furans, mercury, and polychlorinated biphenyls^31^—that harm respiratory health and are linked to asthma and other conditions, including cancer.^32^^–^^34^

Across their entire life cycle, plastics also contributed substantially to climate change, which in itself causes a host of health problems and poses an existential threat to humanity and the planet as we currently know it. Plastics, which originate from fossil fuels, are responsible for up to 6% of global oil consumption, about the same as the aviation industry—an estimate expected to rise to 20% in the next 30 years.^10^ As plastics are burned or break down in the environment, they also release greenhouse gases, including carbon dioxide and methane.

The WHO has maintained that the health risks of microplastic exposure are of relatively low concern.^35^ We are not yet convinced. And, as scientific findings potentially uncover greater impacts, we worry about the difficult challenge of mitigating the consequences of a virtually ubiquitous contaminant. Furthermore, we recognize the global health equity issue that plastics pose beyond just microplastic contamination, and the disproportionate impacts that populations already marginalized by global political and economic structures are made to bear. In addition to individual-level reductions of plastic use, we call for actions that could build a stronger case to policymakers and industrial leaders to abandon fossil fuel-derived plastic products, and curb the unfettered flow of plastic that is projected to double by 2050.^36^

Given that the vast majority of plastic—more than 90%—is not recycled, our dependence on recycling alone is destined for failure. On a global scale, we urgently need to implement other circular economic practices, including redesigning plastic-laden products, reimagining supply chains to decrease packaging and single-use materials, and developing community interventions, especially ones that turn plastic collection and recycling into economic opportunities.^37^^,^^38^ At the same time, we need to continue to develop less harmful plastic alternatives, such as biodegradable and compostable plastics, taking care to ensure that substitutions don’t create additional toxicity, sustainability, or other health concerns, and that the infrastructure required for their disposal, such as commercial composting facilities, is made widely available. Finally, as the problem inevitably intensifies, we also need to identify new methods for managing human exposure to macro-, micro-, and nanoplastics, including through improved sanitation, waste infrastructure, wastewater management, drinking water treatment, air filtration, and potentially novel innovations, such as using polyethylene-metabolizing microorganisms to decompose plastics.^39^^–^^41^

Better understanding the extent of plastic pollution and quantifying its health impacts, especially in vulnerable communities with limited access to waste management, opens up another critical avenue of change. If we’re able to ascertain the real costs of plastic production and waste mismanagement, we can translate this into economic terms, framing plastic pollutants as environmental externalities and designing policies—including carbon and pollution taxation and plastic bans—that disincentivize virgin plastic production and incentivize proper disposal and recycling. Health professionals and other planetary health leaders can also leverage this knowledge to call for shifts in industrial policies and trade agreements that allow for the scaling up of petrochemical plants and the exportation of plastic trash to places least equipped to handle it. The global health community can also help to decrease rampant plastic consumption in the health-care sector, which is also currently estimated to contribute to close to 5% of total global emissions;^42^ contribute to media and social media conversations on plastics and health; help elevate the voices of local communities struggling with plastic pollution and its health consequences; frame plastic pollution in the context of other environmental challenges, including climate change and chemical pollution, to support more comprehensive solutions; and forge interdisciplinary, intersectoral, and community-based collaborations to reduce, divert, and dispose of waste safely.

Solving the global plastic crisis presents a potent opportunity to advance human and planetary health and improve global health equity. As we confront collectively the novel health challenges presented by large-scale environmental issues, the global health community can make a tremendous difference, illuminating win–win strategies that support human thriving and ecological resilience simultaneously. Paying attention to opportunities to speak up and collaborate with environmental scientists, policymakers, community leaders, business partners, economists, and other relevant stakeholders is critical to identifying and implementing solutions to mitigate the harms of plastic and protect the health of the global population, including future generations and those most at risk today.
